# Hemiplegic (unilateral) cerebral palsy in northern Stockholm: clinical assessment, brain imaging, EEG, epilepsy and aetiologic background factors

**DOI:** 10.1186/s12887-020-1955-z

**Published:** 2020-03-12

**Authors:** Elsa Tillberg, Bengt Isberg, Jonas K. E. Persson

**Affiliations:** 1 Department of Clinical Neuroscience, Karolinska Institute, Tomtebodavägen 18 A, 171 77 Stockholm, Sweden; 2 Läkarhuset Odenplan, Odengatan 69, 113 22 Stockholm, Sweden; 3 Department of Clinical Neurophysiology, Karolinska University Hospital, Eugeniavägen 11, 171 76 Stockholm, Sweden

**Keywords:** Hemiplegic cerebral palsy, Aetiologic background, EEG, Epilepsy, Brain imaging

## Abstract

**Background:**

The purpose of this study was to describe clinical presentation, epilepsy, EEG, extent and site of the underlying cerebral lesion with special reference towards aetiologic background factors in a population-based group of children with hemiplegic cerebral palsy.

**Methods:**

Forty-seven children of school- age, fulfilling the SPCE (Surveillance of Cerebral palsy in Europe)-criteria of hemiplegic cerebral palsy, identified via the Swedish cerebral palsy register, were invited and asked to participate in the study.

**Results:**

Fifteen boys and six girls participated. Of the sixteen children born at term, five had no risk factors for cerebral palsy. Two out of five preterm children presented additional risk factors. Debut of motor impairment was observed in the first year of life in sixteen children. Age at diagnosis varied from 2 months to 6 years. Epilepsy was common and associated with grey- and white matter injury.

**Conclusions:**

Recognizing the importance of risk factors for cerebral palsy, any child with these risk factors should be offered a check-up by a paediatrician or a paediatric neurologist. Thereby reducing diagnostic delay. Epilepsy is common in hemiplegic cerebral palsy and associated with grey- and white matter injury in this cohort.

## Background

In a previous study of a subgroup of hemiplegic cerebral palsy, we found a high frequency of epilepsy, almost 50% [1]. We therefore wanted to study a population-based group of hemiplegic cerebral palsy in both term and preterm children.

Cerebral palsy (CP) is an umbrella term for motor impairments, that share features of a non-progressive brain injury, acquired during the early stages of its development [2–4] .These motor impairments can have different aetiologic backgrounds, occurring in the antenatal, perinatal or postnatal period [2–4]. The motor impairment is often associated with visual- and hearing deficits as well as epilepsy and cognitive impairment [2, 3, 5].

Epilepsy is common in cerebral palsy, present in 30–40% of cases [4, 6, 7]. Zelnik found seizures in 35,5% of children, in the subgroup hemiplegia.

Epilepsy is related to the underlying brain lesion [6] . In term children with cerebral palsy, grey matter lesions are more common [6].

The occurrence of neonatal seizures is a risk factor for cerebral palsy [8–10]. Neonatal seizures can be a strong predictor for epilepsy in cerebral palsy [7, 11, 12] . Neurodevelopmental outcome however, is predominantly due to the underlying brain lesion. But clinical studies suggest that the seizures themselves may be detrimental to the developing brain [9].

Few studies address specific EEG-patterns and semiology in cerebral palsy [13]. Most studies of epilepsy in cerebral palsy are based on medical records, or information from cerebral palsy registers. A few direct clinical studies were found [11, 12, 14]. In the studies of Singhi and Gururaj a complete clinical examination was performed on 105, respectively 56 children with epilepsy and cerebral palsy [12, 14].

The cerebral lesion can be classified according to the anatomical site of the brain lesion: Cerebral cortex, pyramidal tract, extrapyramidal systems or cerebellum [3]. The extent of the underlying cerebral lesion must also be recognized [3, 15]. The imaging patterns according to type, as classified by Reid, includes white matter injury, grey matter injury, focal vascular insults and malformations [15].

Children with cerebral palsy, epilepsy and cognitive disability constitute a large patient group in paediatric neurology. An increasing number of register-based studies, address these impairments simultaneously [6, 16, 17].

Due to the heterogeneity of cerebral palsy, it could be useful to study a subgroup, such as hemiplegic cerebral palsy [1, 5, 18] . We wanted to study both clinial presentation, EEG and brain abnormalities found in each child.

## Aetiologic background factors

Since Fiona Stanley published her important book “Cerebral Palsies: Epidemiology and causal pathways”, there is consensus that cerebral palsy results from multiple factors acting along a causal pathway [4, 5].

**Socioeconomic** factors are important [19], especially parents educational level [20–22].

**Gestation: “**Paradox: Very strong relationship between prematurity and risk of cerebral palsy versus the fact that most patients with cerebral palsy are born at term” [4]. Children born before 28 weeks´ gestation, and all preterm children with additional risk factors, such as intracranial haemorraghe, encephalitis, meningitis, seizures and other conditions leading to care in the neonatal unit, are included in the Swedish neonatal society’s high risk group. These children are offered **a** follow up at the neonatal clinic according to the Guidelines from Swedish neonatal society 2015 [23]. Children born at 28–32 weeks are considered an intermediary risk group**.** For term infants, low birthweight, especially infants being small for gestational age (SGA), is a significant risk factor [10, 16].

**Infection**: Cerebral palsy has long been associated with congenital cytomegalovirus (CMV) infection [4]. More recent reports have been published, where CMV-DNA was assessed by PCR in the neonatal screening card [24]. In a study from 2017, 31 (9, 6%) of 401 children with cerebral palsy, tested positive for CMV- DNA.

### Research approach

The purpose of this direct, not register-based, study was to outline clinical presentation, epilepsy, EEG and brain imaging in a population-based group of children with hemiplegic cerebral palsy. The site and extent of the underlying cerebral lesion was also documented. All case records and case histories were reviewed with special reference towards aetiologic background factors. The focus of the study was to identify aetiologic backgrounds- and risk factors, to enable early identification and better care for these children.

## Methods

### Definitions

Cerebral palsy was defined as: Motor impairments, due to a non-progressive brain injury, during the early stages of its development [2, 3]. Hemiplegic cerebral palsy was defined as a unilateral motor impairment.

*Classification.* Participants were classified according to the Surveillance of Cerebral Palsy in Europe (SPCE) guidelines [18]. SPCE encompasses fourteen European centers for the study of cerebral palsy, in eight European countries. The guidelines are used to measure the functional loss associated with cerebral palsy and define criteria for each subtype of cerebral palsy, by distribution and type of motor impairment: Hemiplegic (unilateral) or bilateral, spastic, dyskinetic or ataxic. The timing of the supposed cerebral insult is divided into three categories: pre-, peri- or postnatal.

### Case identification

Children aged seven to 16 years, fulfilling the SPCE-criteria of hemiplegic cerebral palsy, were identified via the Swedish national cerebral palsy register (CPUP). A written survey asking for these children, was also sent to all local rehabilitation-units in northern Stockholm. These children were invited and asked to participate in the study. The caregivers of twenty-one children gave written and informed consent for the children’s participation, and the study was approved by the regional ethics committee.

### Clinical assessment

All case records were reviewed with particular attention to pathogenic factors and pre-peri- or postnatal risk factors for cerebral palsy (18). Common perinatal risk factors are neonatal seizures, asphyxia and cerebral infection. Postnatal head trauma or surgery are important postnatal risk factors. Prenatal risk factors were defined as described by McIntyre [11]. The results of visual- and audition testing were collected. All of the children were examined by the same paediatric neurologist (ET). Parents´ occupation was documented.

Case history included information about gestation, birth-weight, perinatal events, developmental milestones, debut of clinical signs and age at diagnosis. Special focus was laid on early handedness, ie impaired hand function on one side, or delayed or abnormal walking [25]. The neurological examination, as in a previous study [1], consisted of assessment of the cranial nerves, head circumference, muscle tone, motor impairment, tendons reflexes, the Babinski response. Speech was evaluated according to Andersen: Zero indicates normal speech, one indistinct speech, two obviously indistinct speech, three severely indistinct speech, and four no speech [26].

The results of computerized tomography (CT)-scans and or magnetic imaging (MRI) of the brain were collected. For describing the brain morphology revealed by brain imaging we used the classification proposed by Reid [15]. The imaging patterns include white matter injury, grey matter injury, focal vascular insults and malformations. Most studies report only the predominant radiologic alteration. In this study we tried to describe the different radiologic abnormalities in each child. All available MRI’s and or CT-scans of the brain, were collected and reviewed by the same experienced radiologist.

Epilepsy was diagnosed according to ICD, by the paediatric neurologist at the Karolinska University Hospital, before study comenced. Epilepsy was classified according to the latest criteria of the International league against epilepsy, 2017 [27]. Focal seizures with or without secondary generalization were categorized in the same subtype [7]. Electroencephalogram (EEG) was recorded according to the 10–20 system, 20 min´ registration with alert patient. All EEG: s from the study and earlier EEG:s from the case records, were viewed by an experienced neurophysiologist (JP) together with the paediatric neurologist (ET).

All participants were offered a cytomegalovirus DNA assessment in their neonatal screening card.

## Results

### Clinical assessment

The results are summarized in Table 1. Clinical presentation.

**Table 1:** Clinical presentation

Pat nr gender	Side of motor impairment/ Age at examination	Mothers/Fathers profession	Gestation/ Birth weight	Perinatal events	Debut motor impairment/ age at diagnosis	Neurological examination/ dorsiflexion of the great toe (Babinski response) impaired side	Hearing Speech observation: 0 1 2 3 4 (Andersen et al. 2008)	Vision/ eye function	Postnatal events/ other
1.M	right/ 14 years	Teacher / security gard	40 weeks / 4015 g	asphyxia neonatal seizures	1 year fine motor impairment/ 1,5 year	Tendon reflexes++, / Babinski response ambiguous	bilateral sensory neuronal hearing loss, diagnosis at age 6 years! 0 normal	normal	
2.M	left/ 8 years	kitchen assistant cook	40 weeks/ 3855 g	–	1 year fine motor impairment/ 1,5 years approximately	weak depressor of the corner of the mouth (N VII) and shoulder (N XI) reflexes ++ / Babinski positive	Sensory neuronal hearing loss 0 normal	myopia, crowding	
3.M	left/ 10 years	primary school teacher/ civil engineer	32 weeks/ 1354 g SGA, small for gestational age	preeclampsia caesarean section	7 months/ 7 months	squint, weak corner of mouth(NVII) and shoulder (NXI) tongue deviation to the left (XII) reflexes ++ / Babinski positive	normal 1 indistinct	central visual impairment, crowding	
4.M	right/ 10 years	secondary school teacher/ civil engineer	42 weeks/ 3150 g (− 1 SD)	induced labour. Fast parturition	4 months/ 8 months	weak shoulder (NXI), reflexes ++ / Babinski positive	normal 0	normal	
5.M	right/ 8 years	accountant/ civil engineer	41 weeks/ 3200 g (− 1SD)	–	6 months /11 months	weak corner of mouth (NVII) and shoulder (NXI) / Babinski positive	normal 1 indistinct	normal	
6.M	right/ 12 years	preschool teacher/ primary school teacher	3400 g/ 40 weeks	–	4 months 7 months	reflexes + /Babinski negative	normal 0	normal	
7. F	right/ 10 years	occupational therapist/ secondary school teacher	35 weeks/ 4328 g (Large for gestational age)	maternal diabetes	1,5 years 6 years, by paediatric neurologist!	weak corner of mouth (N VII), reflexes + /Babinski positive	normal 0	normal	
8.F	right/ 13 years	secondary school teacher/ electrician	4050 g/ 40 weeks	Rapid parturition	1,5 years / 2 years	reflexes ++/ Babinski ambiguous	normal 0	normal	
9. M	left/ 10 years	school assistant/ unknown	33 weeks/ 1842 g (− 1 SD)	–	1 year/ 1 year symptoms after accident	weak corner of mouth(N VII) and shoulder(N XI), reflexes ++ /Babinski ambiguous	normal 0	homonym, lower quadrant defect left	car accident age 1 year. Op subarachnoid haemorraghe
10. F	right/ 10 years	sales person/ programmer	3500 g/ 38 weeks	caesarean section because of maternal diabetes	6 months 1 year	weak shoulder (N XI), reflexes ++ / Babinski positive	normal 0	normal	
11-M	right/ 10 years	social scientist/ engineer	41 weeks/ 4485 g (large for gestational age)	caesarean section IVF convulsions at 18–24 h age	1 year 1,5 year	intermittent strabismus, reflexes +/ Babinski positive	normal 0	Heterotopia hypermetropi	
12.F	left/ 15 years	health care assistant/ chief product officer with university degree	39 weeks/ 2600 g (SGA)	Twin II caesarean section	2 months 2 months	nystagmus, strabismus, weak corner of mouth (VII) and shoulder (XI),reflexes ++/ Babinski positive	normal 1	homonym hemianopia left	Generalized epilepsy at 2 months. Hemi-megaencephali, peri- insular hemisphere-ektomi age 1 year/ bilateral nystagmus
13.M	right/ 7 years	economist/ economist	32 weeks/ 1900 g	–	1,5 year 5 years	reflexes ++/ Babinski ambiguous	normal 0	normal	
14. F	left/ 14 years	authorized accountants both	29 weeks/ 1500 g	–	5 months 8 months	reflexes ++ Babinski positive, weak right corner of mouth	normal 0	normal	
15.M	right/ 11 years	no occupation entrepreneur	41 weeks/ 3650	prolonged labour.	1 year 1 year	strabismus, tongue deviation (XII),weak shoulder (XI), reflexes ++/ Babinski positive	Sensory neuronal hearing loss right 0	homonym quadrant defect right	
16. M	right/ 10 years	preschool teacher/ researcher at university	40 weeks/ 3700 g	–	6 months/ 6 months	weak corner of mouth, impaired wrinkling of forehead (VII lower motor neuron), weak shoulder (XI), reflexes++/Babinski ambiguous	normal 0	total hemianopia right	traumatic epidural hematoma age 6 months
17.M	left/ 13 years	solicitor/ solicitor	40 weeks/ 3980 g	–	14 months/ 2 years	reflexes. ++ / Babinski positive	normal 0	normal	
18.F	right/ 9 years	preschool teacher/ painter	40 weeks/ 3600 g	–	3 months/ 8 months	reflexes ++/ Babinski positive, weak left corner of mouth	normal 0	normal	
19.M	right/ 15 years	human resources/ officer	37 weeks/ 3375 g	caesarean section because of prenatally diagnosed hydrocephalus	3–6 months	strabismus, weak shoulder (XI) reflexes ++ / Babinski positive	normal 0	normal	Neonatal shunt operation
20.M	left/ 12 years	personal assistant /taxidriver	40 weeks 2990 g (− 1,5 SD)	–	1,5 years/ 2 years	reflexes normal Babinski positive	normal 0	normal	
21.M	right/12 years	Headmaster/ shop assistant	41 weeks 3900 g	–	6 months 2 years	weak corner of mouth (VII), reflexes ++ Babinski positive	normal 0	normal	

Forty-seven children fulfilling the SPCE-criteria of hemiplegia were identified and invited to participate in the study. The majority of the 21 children who consented to participate in the study were boys, fifteen. Only six girls took part in the study. Five children were born preterm, three males and two females. Right-sided hemiplegia was present in fourteen and left-sided in seven children. Only one child was dyskinetic, the others were spastic. Cranial nerves were affected in 14 of the 21 children. Strabismus was found in five, hemianopia, or quadrant defect in four children. Sensory neuronal hearing loss occurred in three children, indistinct speech was observed in two.

Out of the forty-two parents, seventeen had higher educational level. The others had mainly lower educational level. Professions were variable.

### Risk factors

Five children were born preterm, four of them before 34 weeks´ gestation. Five children were delivered by caesarean section, one because of prenatally diagnosed hydrocephalus. Parturition was rapid in two children, prolonged in one. Diabetes was present in two mothers. Two children were small for gestational age (SGA) and two were large for gestational age (LGA). Neonatal seizures occurred in two of the children, for one concomitantly with asphyxia. One child, caesarean section, because of prenatally diagnosed hydrocephalus, received a ventriculo-peritoneal shunt in the neonatal period. One child was born preterm and suffered postnatal skull trauma, one term child suffered postnatal skull trauma and another underwent postnatal hemisphere-ektomi. Only five of the sixteen children born at term, had no pre- or perinatal risk factor for cerebral palsy. In all only five of the 21 children (24%) had no pre-, peri- or postnatal risk factor for cerebral palsy.

### Developmental milestones

Early handedness was present in more than 90% of the cases, 20 out of 21 children. It was noted from 3 months to 1 year of age in 16 out of 21 children. Abnormal or delayed walking was present in eight children.

### Brain imaging findings

The results are summarized in Table 2. Brain imaging findings and Table 3. Epilepsy and brain imaging.

**Table 2:** Brain imaging findings

Child No# Gender	MRT/CT	Clinical side	Gestation < 34 weeks	Gestation > = 34 weeks	Imaging pattern				Miscellaneous	Localisation Extent and details of brain lesion
					Malformation	White matter injury	Grey matter injury	Focal vascular insult		
1.m	MRT	Right		+		+	+		Affection of left pyramid tract, basal ganglia, cerebellum	Left Volume loss in white matter and cortex
2.m	MRT	Left		+	+					Right Polymicrogyri whole hemisphere
3.m	MRT	Left	+			+				Bilateral PVL (periventricular leukomalacia) ill defined
4.m	MRT/CT	Right		+				Infarction of a.cerebri media, almost total		Left Extensive loss of parenchyma in frontal and parietal lobe
5.m	CT	Right		+		+				Bilateral, but predominantly left PVL
6.m	–	Right								No examination
7.f	MRT	Right		+		+				Bilateral Slight PVL
8.f	MRT	Right		+		+				Left, Slight PVL
9.m	CT	Left	+			+	+		Traffic accident at age 1 year	Right Volume loss frontotemporally 43 × 46 mm
10.f	MRT	Right		+				Infarction of a cerebri media	Wallerian degeneration	Left Extensive
11.m	CT	Right		+				Infarction a.cerebri ant+posterior		Left Extensive
12.f	MRT	Left		+	+				Hemi megaencephali	Right Extensive Peri-insular hemisphere ectomi at age 1 year
13.m	CT	Right	+			+	+			Left Thin spalt extends from lateral cerebral surface medially
14.f	CT	Left	+			+				Right Moderate PVL
15.m	MRT/CT	Right		+		+	+		Pyramidal tract loss Affection of radiatio optica	Left Extensive parenchymal defect frontal-parietal + temporal lobe
16.m	MRT/CT	Right		+				Post traumatic epidural hematoma		Left Extensive loss of most of hemisphere+ small lesion in cerebellar lobe Post traumatic epidural hematoma at age 6 months
17.m	MRT	Left		+		+				Right PVL superior of lateral ventricle
18.f	MRT	Right		+		+				Left PVL
19.m	MRT/CT	Right		+				peri-ventricular hemorraghic infarction		Left Extensive sequelae with dilatation of the left lateral ventricle
20.m	MRT	Left		+	+				Susp lesion of pyramidal tract	Right Polymicrogyri in frontal+ parietal lobes
21.m	MRT	Right		+					Left thalamus smaller	Left hemisphere smaller Volume loss of central white matter

*PVL* (Periventricular leukomalacia)

*MRT* (Magnetic Resonance Tomography of the brain)

*CT* (Computerised Tomography of the brain)

**TABLE 3:** Epilepsy and Brain Imaging

Nr	Brain imaging findings	Epilepsy	
1	White Matter Injury (WMI) + Grey matter Injury (GMI)	neonatal convulsions focal seizures	
2	GMI	–	
3	WMI	–	
4	WMI + GMI focal vascular insult	focal seizures	
5	WMI	–	
6	no examination	–	
7	WMI	–	
8	WMI	–	
9	WMI + GMI	focal seizures	
10	WMI + GMI+ focal vascular insult	focal seizures	
11	WMI + GMI+ focal vascular insult	neonatal convulsions	
12	WMI + GMI hemi-megaencephali	generalized epilepsy	
13	WMI+ GMI	focal seizures	
14	WMI	–	
15	WMI+ GMI	focal seizures	
16	WMI + GMI + focal vascular insult	generalized seizures	
17	WMI	–	
18	WMI	–	
19	WMI + GMI focal vascular insult	focal seizures	
20	GMI	–	
21	WMI+ GMI	–	

**Fig. 1:**
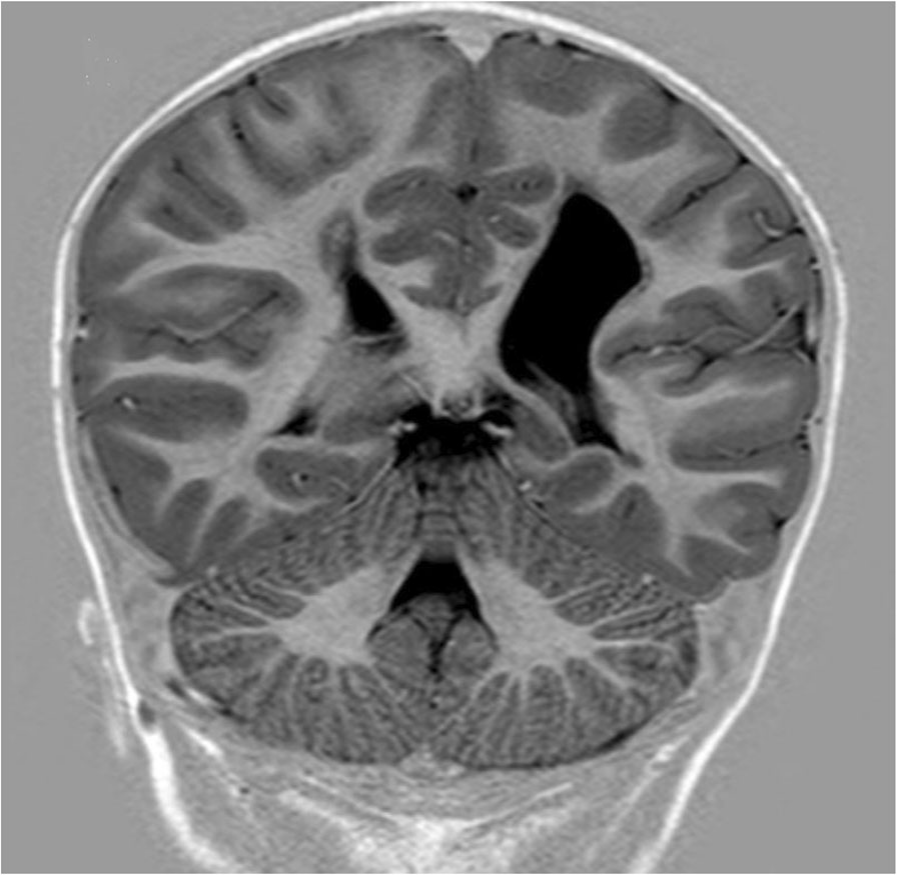
White matter lesion. Sequelae after periventricular leucomalacia in left parietal lobe, but also in right cerebral hemisphere. Coronal image

**Fig. 2:**
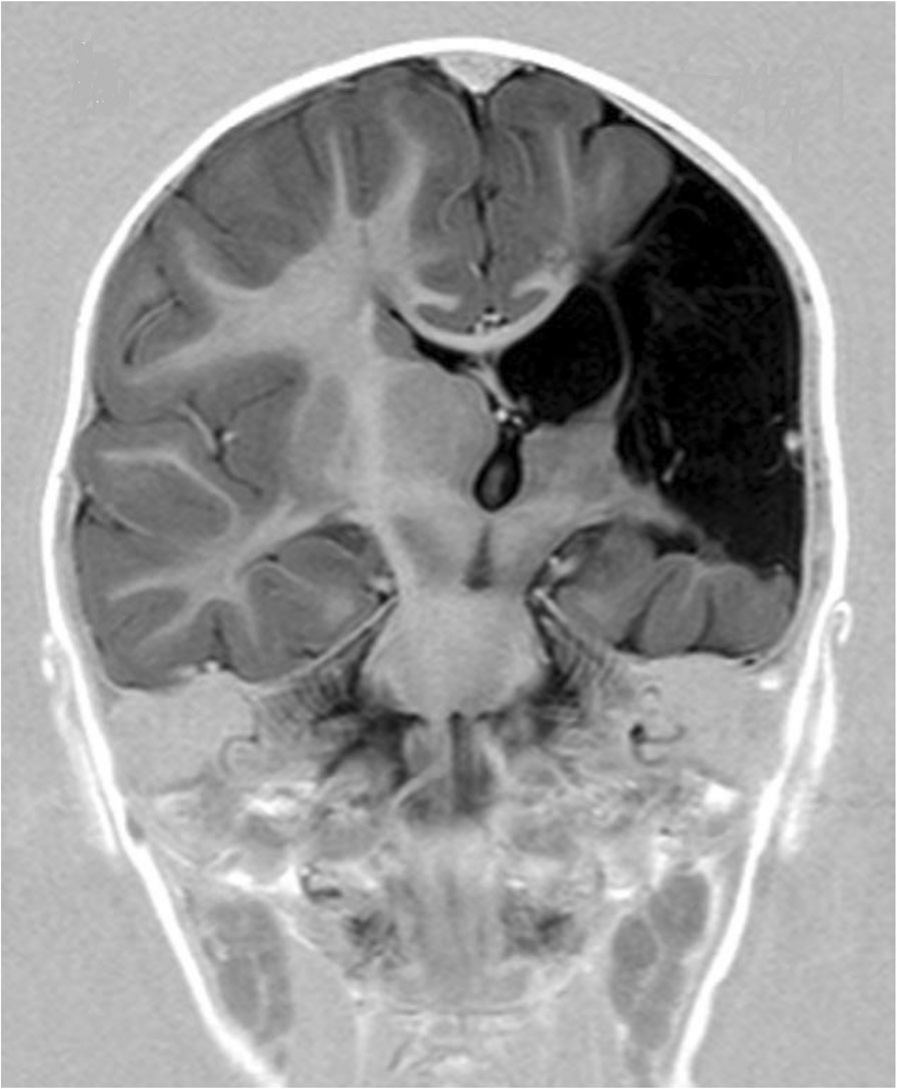
Sequelae after vascular insult of left middle cerebral artery with major volume loss. Coronal image

**Fig. 3:**
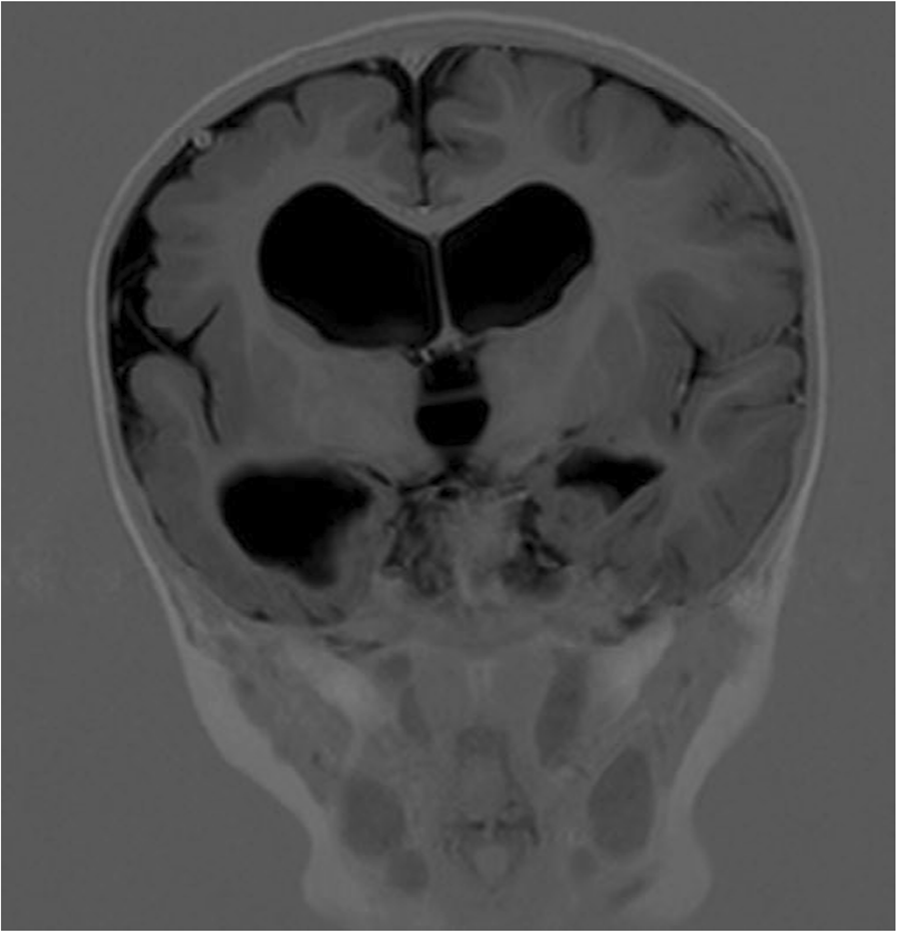
Cortical malformation, polymicrogyri, in right frontal lobe. In addition dilated ventricles. Coronal image

**Fig. 4:**
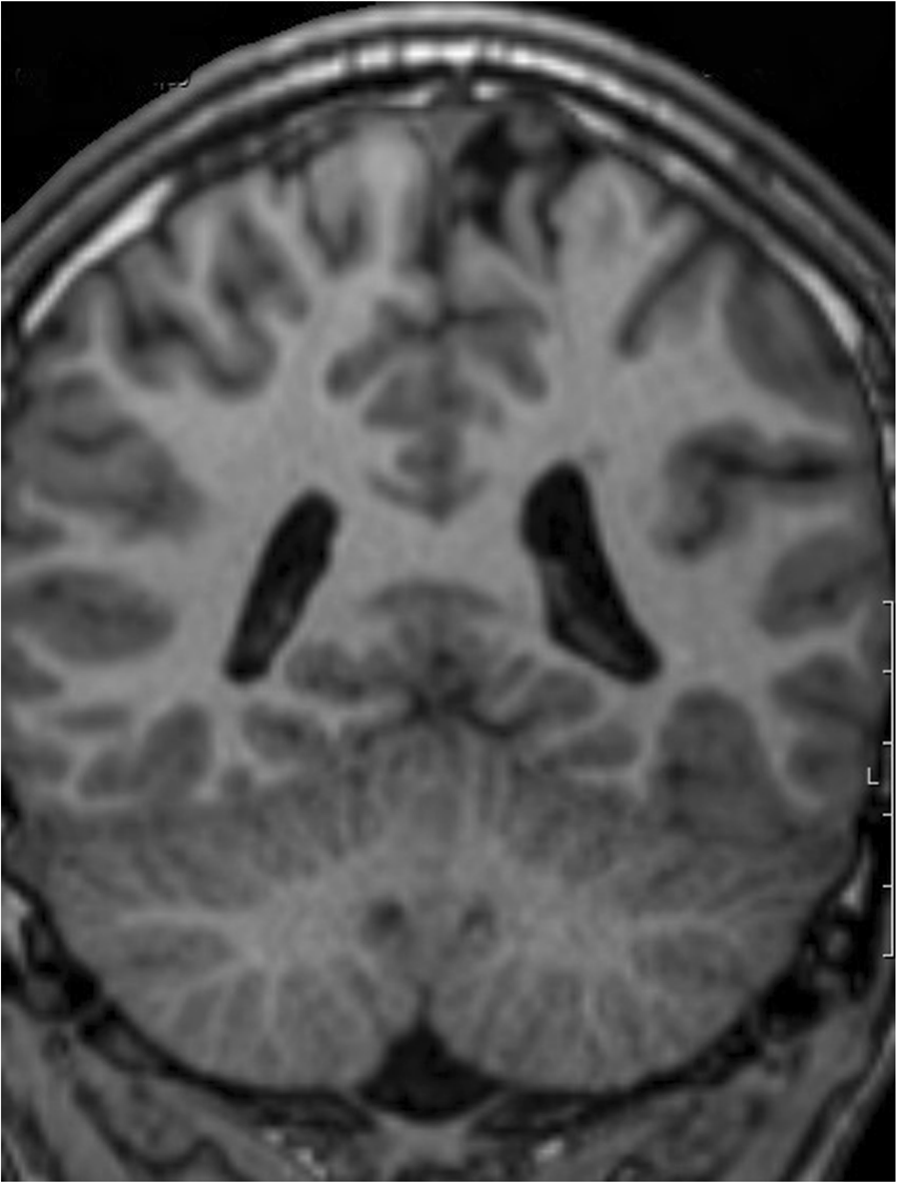
Combined lesion in white matter and cortex. Coronal image

For twenty of the twenty-one participants a MRI and or CT-scan of the brain was available. Examinations were performed from the neonatal period up to 17 years of age. Four of the twenty children with examinations, were born before 34 weeks´ and sixteen at, or after 34 weeks´ gestation. Left-sided lesion was present in eleven, right-sided in six and bilateral in three children. Seven children showed white matter injury only, two preterm and five term children, for an example see Fig. 1. Grey matter injury only, was not found in any of the children. The five children with focal vascular insults, all had extensive lesions that affected both white and grey matter, see Fig. 2. Malformation was found in four children. Polymicrogyri, which mainly affects cortex, was present in two of them, for an example see Fig. 3. White- and grey matter combined, was present in four children, two term and two preterm, for an example see Fig. 4. The extent of the lesion varied from slight periventricular leucomalacia (PVL) in child No. seven and No. eight, to loss of most of the left hemisphere in child No. sixteen. Summarized in Table 2, Brain imaging findings and Table 3, Epilepsy and brain imaging.

### Epilepsy, neonatal seizures and EEG

Two of the children, who later developed epilepsy were born preterm. One of them, child No 9 had focal seizures at 1 year of age, after a traffic accident. The other child developed focal seizures, the most common seizure type in this study.

Neonatal seizures occurred in two children. Nine children developed epilepsy. Of these, seven children developed focal seizures and two generalized seizures. Epilepsy onset was from one to 12 years of age. Diagnosis was made at the neuropaediatric unit, Karolinska University Hospital. Seizures resolved at three to 12 years of age. Active epilepsy was still present in four children out of nine, two were seizure free with AED. Initial treatment was mostly with Lamotrigin, Hydroxykarbamazepin or Levetiracetam. Resistance to drug theraphy was found in three Children. Child No. 16 tried ketogen diet, without success. None of the children were identified with epileptic encephalopathy. Focal abnormalities in the EEG were the main findings, as summarized in Table 4, EEG and epilepsy. Ten children had normal EEG: s.

**Table 4:** EEG and epilepsy

Nr.	Age at exam	Gender	Motor impairment	EEG findings	Other Events	Epilepsy Seizure type Debut at age	Active	Resolved at age Seizure free with AED
1	14	Male	Right	Mild episodic abnormity maximum left temporally		neonatal convulsions focal seizures 2 years	–	treatment neonatally to 6 months treatment from 2 to 3 years
2	8	Male	Left	Moderate generalized abnormality left dominance		–	–	
3	11	Male	Left	Normal EEG		–		
4	12	Male	Right	No study EEG. Clinical EEG at seizures onset focal left epileptiform activity		Focal seizures 12 years	+	
5	9	Male	Right	Normal EEG		–	–	
6	12	Male	Right	mild generalized abnormality		–	–	
7	10	Female	Right	Mild, mainly episodic bilateral abnormality right dominance		–		
8	13	Female	Right	Normal EEG		–	–	
9	10	Male	Left	Focal abnormality temporo-parieto-occipitally right. Bilateral reduced alpha-activity	Traffic accident at 1 year	Focal seizures 1 year		5 years
10	10	Female	Right	Normal EEG		focal seizures > 12 years 2 years after study examination	–	
11	10	Male	Right	Normal EEG		Short focal neonatal convulsions	–	
12	15	Female	Left	Continuous generalized epileptiform discharges with right dominance	Right-sided hemispherectomi	generalized epilepsy 2 months	+	Resistant to AED
13	7	Male	Right	Normal EEG		Focal seizures 4 years	+	
14	14	Female	Left	Normal EEG		–		
15	11	Male	Right	Mild generalized abnormality with left dominance with sparse amount of epileptiform discharges left frontal and medial		Focal seizures 4 years	–	Seizure free with AED
16	9	Male	Right	Moderate amount of epileptiform discharges left hemisphere fronto-centrally and/or fronto-temporally	Head trauma at 6 months, epidural hematoma	Generalized seizures since trauma	+	AED therapy resistant
17	13	Male	Left	Normal EEG		–		
18	9	Female	Right	Mild to moderate amount of generalized slow activity with slight left-sided dominance		–		
19	15	Male	Right	Somewhat increased amount of slow activity over the left hemisphere. No distinct epileptiform activity.		Focal seizures 3 years.		No seizures since age 9 years with AED
20	12	Male	Left	Normal		–		
21	12	Male	Right	Normal		–		

### Brain imaging and epilepsy

All children who developed neonatal seizures, or later on epilepsy, showed both white and grey matter injury. Patient No. 16 with the most extensive brain lesion, consequently had the most serious seizures: Drug therapy resistant generalized seizures. The others responded to therapy. The grey- and white matter injury in three children, was a part of a focal vascular insult. See Table 3.

CMV- DNA was negative for the eleven children tested.

## Discussion

Less than 50% of the eligible subjects participated, which may have biased the results. Probably the children with most problems, consented to participate. This may be due to the sparse attention, which is offered in routine health care follow up.

In agreement with many other studies, 70 % of the participants in this study were male [28] .

In Stockholm, Sweden, only children included in the high risk group are considered for special follow up by a neonatologist or neurologist. This high risk group encompasses major perinatal risk factors such as asphyxia, neonatal seizures, cerebral haemorraghe, hypoxic- ischaemic encephalopathy, sepsis etc., leading to care in the neonatal unit. Birth before 28 weeks´ gestation and/ or children being small for gestational age are also included in the high risk group [23]. Children without major perinatal risk factors will be examined by a general practitioner at the child health center.

Most patients with cerebral palsy are born at term [4]. In this study, term children comprised 70 %. In “A systematic review of risk factors for cerebral palsy in children born at term in developed countries”, McIntyre found ten consistent risk factors for cerebral palsy in term children: Placental abnormalities, major and minor birth defects, low birth weight, meconium aspiration, instrumental/ emergency caesarean delivery, birth asphyxia, neonatal seizures, respiratory distress syndrome, hypoglycaemia and neonatal infection. However, other risk factors such as maternal disease or being large for gestational age were not statistically significant. But these risk factors may work together along a causal pathway [10]. Thirteen of the sixteen term children had consistent risk factors for cerebral palsy, according to McIntyre [10]. Three of them had minor risk factors, such as rapid parturition and prolonged labor, see Table 1 clinical presentation. In all only five of the 21 children had no pre-, peri- or postnatal risk factors for cerebral palsy.

Motor impairment as observed by parents was evident at age 1 year or before. However diagnosis, was given only between age one and a half to 6 years! Early handedness was present in 90% of the cases. In Japan all children can meet a paediatrician for regular care.

Although epilepsy is present in 30–40% of cases, not many recent studies of epilepsy in cerebral palsy were found [6, 8, 9, 11, 13]. In this cohort of 21 children, nine (40%) developed epilepsy. All had both grey and white matter injury. Of these, five had a focal vascular insult, that affected both white and grey matter. In the study published by Reid 2015 the prevalence of epilepsy was highest where there was generalized cortical-subcortical involvement and white matter loss [30]. (In the study by) Legault [31] found epilepsy more frequent in cerebral palsied children with cerebral vascular accident, or deep brain injury. The size of the injury is also important. The seizures of the child, with the most extensive injury, were drug therapy resistant. Very few clinical studies, if any describe EEG, epilepsy in cerebral palsy. Most studies are register based.

## Conclusions

Pre-, peri- or postnatal risk factors for cerebral palsy, present in sixteen out of twenty-one hemiplegic children in this study, are important for early case identification. Parents often observe the child’s assymetric hand function during the first year of life. To avoid diagnostic delay, any child with risk factors for cerebral palsy and or impaired hand function, should be offered a check-up by a paediatrician or a paediatric neurologist.

A combined grey- and white matter injury, often as a part of a focal vascular insult, was associated with epilepsy in this cohort. EEG can improve the understanding of epilepsy in cerebral palsy.

## Data Availability

The protocols and dataset generated during the study, will be available to view upon reasonable request by contacting the corresponding author.

## Abbreviations

CMV: Cytomegalovirus
CP: Cerebral Palsy
CPUP: Swedish national cerebral palsy register
CT: Computerized tomography of the brain
EEG: Electro EncephaloGram
LGA: large for gestational age
MRI: Magnetic resonance imaging of the brain
PCR: Polymerase chain reaction
PVL: Periventricular leukomalacia
SGA: Small for gestational age
SPCE: Surveillance of Cerebral Palsy in Europe
